# cpxDeepMSA: A Deep Cascade Algorithm for Constructing Multiple Sequence Alignments of Protein–Protein Interactions

**DOI:** 10.3390/ijms23158459

**Published:** 2022-07-30

**Authors:** Zi Liu, Dong-Jun Yu

**Affiliations:** School of Computer Science and Engineering, Nanjing University of Science and Technology, Nanjing 210094, China; liuzi189836@163.com

**Keywords:** protein–protein interactions, protein complex, multiple sequence alignment, genomic distance, phylogeny information, STRING interaction network, sequence coevolution analysis

## Abstract

Protein–protein interactions (PPIs) are fundamental to many biological processes. The coevolution-based prediction of interacting residues has made great strides in protein complexes that are known to interact. A multiple sequence alignment (MSA) is the basis of coevolution analysis. MSAs have recently made significant progress in the protein monomer sequence analysis. However, no standard or efficient pipelines are available for the sensitive protein complex MSA (cpxMSA) collection. How to generate cpxMSA is one of the most challenging problems of sequence coevolution analysis. Although several methods have been developed to address this problem, no standalone program exists. Furthermore, the number of built-in properties is limited; hence, it is often difficult for users to analyze sequence coevolution according to their desired cpxMSA. In this article, we developed a novel cpxMSA approach (cpxDeepMSA. We used different protein monomer databases and incorporated the three strategies (genomic distance, phylogeny information, and STRING interaction network) used to join the monomer MSA results of protein complexes, which can prevent using a single method fail to the joint two-monomer MSA causing the cpxMSA construction failure. We anticipate that the cpxDeepMSA algorithm will become a useful high-throughput tool in protein complex structure predictions, inter-protein residue-residue contacts, and the biological sequence coevolution analysis.

## 1. Introduction

Proteins play crucial roles in almost all biological processes in cells. These important biomolecules, particularly proteins, accomplish their roles by using intermolecular interactions, such as the identity, dynamics, and specificity of protein interactions [1,2]. Experimental screens have identified tens of thousands of protein–protein interactions (PPI) or protein complexes, and structural biology has provided detailed functional insight into select 3D protein complexes. However, the structures of many protein complexes are unknown, and there is still little, or no, 3D information for a significant percentage of currently known PPIs or protein complexes in bacteria, yeast, and humans [3,4]. The structures of many essential PPI complexes, including those bound with the cell membrane, are difficult, if not impossible, to solve using the current techniques. The computational approach has therefore become an increasingly important means to obtain protein complex structures, especially for large-scale protein complex structure modeling [5].

With the rapid growth in our knowledge of genetic variation at the sequence level, there is increased interest in linking sequences with the change in molecular interactions. However, the current experimental approaches cannot meet the demand for residue-level information on these interactions. Recent work has demonstrated the accuracy of co-evolution-based contact prediction for monomeric proteins using global statistical models [6,7,8]. The chain’s multiple sequence alignment (MSA) is the fundament of the quantified coevolution. MSA provides more information by showing conserved regions and motifs of structural and functional importance within the protein family. Furthermore, the MSA is an essential part of protein structure prediction [9], protein contact map [10], second structure feature [11], ligand-binding site prediction [12], homologous templates [13], gene ontology [14], phylogenetic analysis [15], and many other valuable procedures in sequence research [16]. Therefore, many MSA construct methods have been developed, such as BLAST [17], HHblits [18] from the HH-suite [19], and Jackhammer and HMMsearch tools from the HMMER suite [20], MetaPSICOV2 [21], and DeepMSA [22].

**Figure 1:**
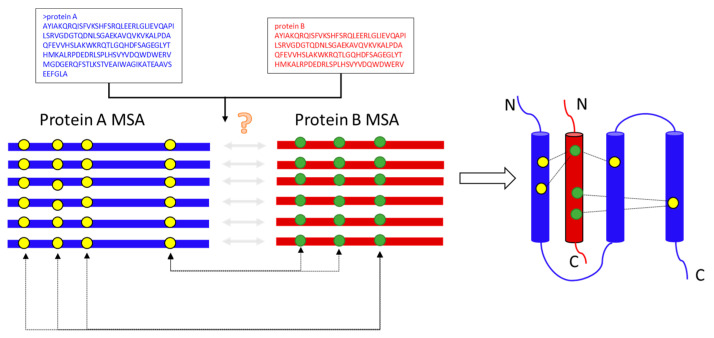
The flowchart of the building cpxMSA. The two circles (yellow and green) connected by double arrow lines indicate sites of coevolution (**left**) to identify evolutionary couplings between co-evolving inter-chain residue pairs (**right**).

In contrast to the extensive work on monomeric proteins, little is known about the utility of such statistical models for predicting protein–protein interactions or protein complexes. Coevolution is at the basis of many modern computational techniques for characterizing protein−protein interactions. Therefore, as shown in Figure 1, how to build the multiple sequence alignment (MSA) of the protein–protein interaction or protein complex is an important issue that needs to be addressed. EVcomplex [3], Gremlin-Complex [23], and ComplexContact [4] are based on the genomic distances to build protein complex multiple sequence alignments. ComplexContact [4] also creates protein complex multiple sequence alignment by using a phylogeny-based method.

Although the above methods developed the genomic-based and phylogeny-based methods to generate protein complex multiple sequence alignments, few standalone pipelines/programs exist that efficiently generate sensitive protein complex MSAs from the input protein complex sequences; hence, there was an urgent need to address this issue. Inspired by the protein monomer MSA algorithm DeepMSA, we developed and released cpxDeepMSA, a new open-source program to construct deep and sensitive protein complex MSAs by merging sequences from three different strategies through a hybrid homology–detection approach.

## 2. Results and Discussion

### 2.1. Evaluation

We evaluated our cpxMSA method for contact prediction using the state-of-the-art programs CCMpred [24] and trRosettaX [25,26]. We calculated the accuracy of the top 50, 20, 10, 5, and top L/k (k = 5, 10, 20, 50) predicted contacts where L is the total length of the two protein chains. The prediction is defined as the percentage of correctly predicted contacts among the top predictions.

### 2.2. cpxDeepMSA Increases Protein Complex Contact Prediction Accuracy

The genomic-, phylogeny-, and STRING-based methods for cpxMSA construction complement each other. Generally, for prokaryotic species, the genome-based method works better, and for eukaryotes, our phylogeny-based method works better, as shown in Table 1, which was tested on the PDB100 (of 100 heterodimers) database by using the predictor trRosettaX (with defaults: “predict.py -i input.a3m -o output.npz -mdir./model_res2net_202012”). The benchmark PDB100 was extracted from a Protein Data Bank (PDB) [27], and the sequence identity cutoff in the benchmark was 40%. The results indicate that for the cpxMSA construction method there is little difference between genomic-based (stage 1) and phylogeny-based (stage 2) and all of them are better than STRING-based (stage 3). The MSA from cpxDeepMSA outperforms the other three MSAs for contact prediction. For instance, when using the MSA from cpxDeepMSA, the precision for the top five contacts was 0.673; this was 58.7%, 16.6%, and 99.1% higher than that of the MSA from genomic-, phylogeny- and STRING-based, respectively.

To further investigate the effectiveness of cpxDeepMSA, we list in Table 2 the comparison of the contact map prediction results of cpxDeepMSA and RoseTTAFold (RF) MSA [28] on the Baker’s dataset [23]. We used CCMpred with parameters “CCMpred input.aln output.mat -n 100 -e 0 -A” to detect the co-evolution on each alignment. Significant improvement of cpxDeepMSA was shown for the contact map prediction over RF MSA on the predictor CCMpred and trRosettaX. In comparison, the corresponding precision for the *top* 10 contacts of RF MSA by CCMpred and trRosettaX were 0.4% and 51.9%, respectively. cpxDeepMSA achieved precision for the *top* 10, with 38.5% and 55.2%, which were 9225.0% and 6.4% higher than RF MSA with CCMpred and trRosettaX, respectively.

### 2.3. Web-Server and User Guide

To enhance the value of its practical applications, the web server for cpxDeepMSA was established. Below, we further give a step-by-step guide on how to use the web server to obtain the desired results.

#### 2.3.1. Server Input

**Figure 2:**
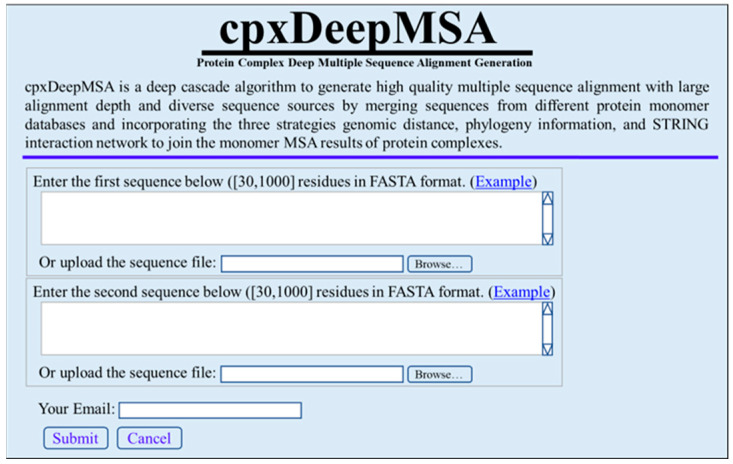
A semi-screenshot showing the top page of the cpxDeepMSA web server at https://zhanggroup.org/cpxDeepMSA/.

Opening the web server at https://zhanggroup.org/cpxDeepMSA/, you will see the top page of the cpxDeepMSA on your computer screen, as shown in Figure 2. The input to the cpxDeepMSA server involves two single-chain amino acid sequence files in FASTA format. After submitting a job, a URL link with a random job ID is generated, allowing the user to check the results and keep the data private. The user must provide an email address when submitting a job, and the server will automatically send a notification email with a link to the results page upon the job completion.

#### 2.3.2. Server Output

**Figure 3:**
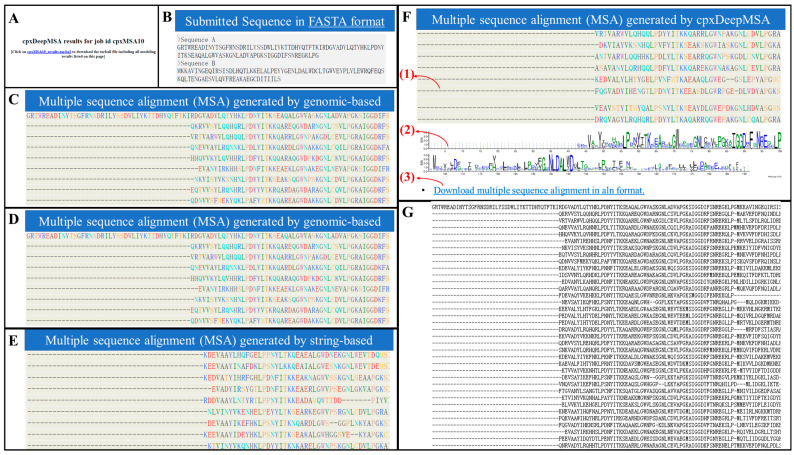
Illustration of the cpxDeepMSA server output, including (**A**) a summary of the multiple sequence alignment results; (**B**) summary of the user input; (**C**–**F**) protein complex multiple sequence alignment-generated-based string, genomics, phylogeny, and cpxDeepMSA, respectively; (**G**) multiple sequence alignment file.

The cpxDeepMSA results page consists of seven sections: (i) A summary of the multiple sequence alignments and sequence analysis compressed package files (Figure 3A), (ii) a submission including a query sequence (Figure 3B), (iii)–(vi) protein complex multiple sequence alignment-generated-based string, genomics, phylogeny, and cpxDeepMSA, respectively (Figure 3C–F), (G) the multiple sequence alignment file (Figure 3G). As an illustration, Figure 3 presents an example from the conformationally-strained, circular permutant of barnase (PDB ID: 3da7) to explain section vi of the results page.

Section vi (Figure 3F) shows the cpxMSA generated by cpxDeepMSA, which lists three parts, (1), (2), and (3). For (1), which shows the cpxMSA on the page, users can drag or zoom in on the table to check the cpxMSA. Additionally, (2) presents the sequence analysis of cpxMSA using the software WebLogo 3.6 [29]. In the last subsection, (3), an aln-formatted file can be downloaded by clicking on the link at the bottom table.

## 3. Materials and Methods

### 3.1. cpxDeepMSA Pipeline for MSA Construction

**Figure 4:**
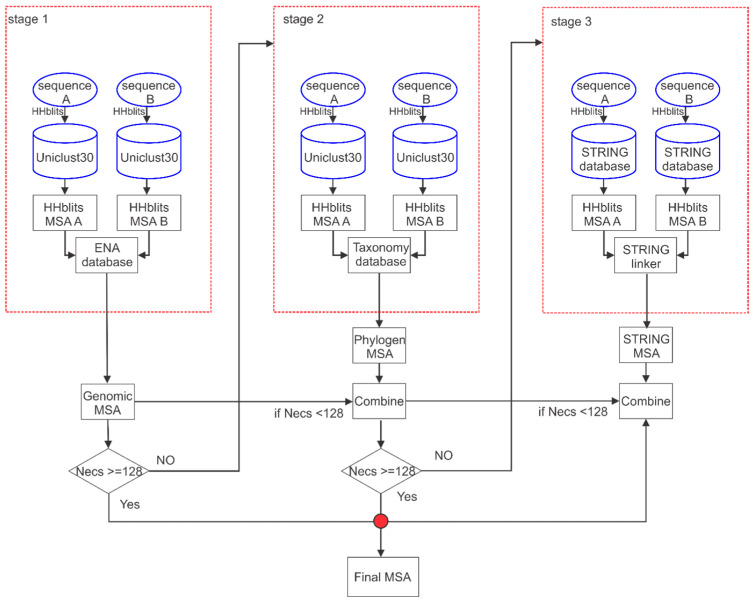
The flowchart of cpxDeepMSA. Three stages of MSA generations were performed consecutively using sequences from the HHblits search through Uniclust30 and pairing with genomic distance (first column), phylogeny information (second column), and the STRING interaction network (third column).

Figure 4 shows a complex pipeline that can be divided into three stages, which correspond to searching two protein sequence databases, Uniclust30 [30] and STRING [31], combining the HH-suite [19] program, and through three matching databases, ENA [32], Taxonomy [33], and STRING linker [31].

**Figure 5:**
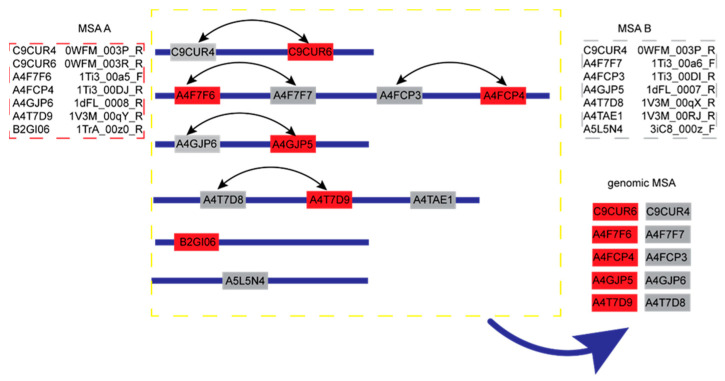
An example of the genomic-based MSA concatenation.

Stage 1. First, download the Uniclust30 (version: 2018_08) [30] protein monomer sequence database from the whole genome data of the protein monomer sequence. Secondly, use the multiple sequence alignment software HHblits (with the parameters “-diff inf -id 99 -cov 50 -n 3”) from the HH-suite 2.0.16 program to search the protein sequence database Uniclust30 for query sequence A and sequence B, respectively. Additionally, obtain the multiple sequence alignment information MSA_A and MSA_B of the protein monomer sequence, respectively. Third, compare the results MSA_A and MSA_B in the genome database (ENA), and obtain the gene information MSA_A_gene and MSA_B_gene of the multiple sequence alignment results. Fourth, according to the gene distance Δgene of the two protein sequences i and j with the same gene in the MSA_A_gene and MSA_B_gene, if 1≤Δgene≤20, connect the protein sequence i and j. Finally, according to the above steps, construct a multiple sequence alignment (MSA) of the protein complex based on gene distance, as shown in Figure 5.

**Figure 6:**
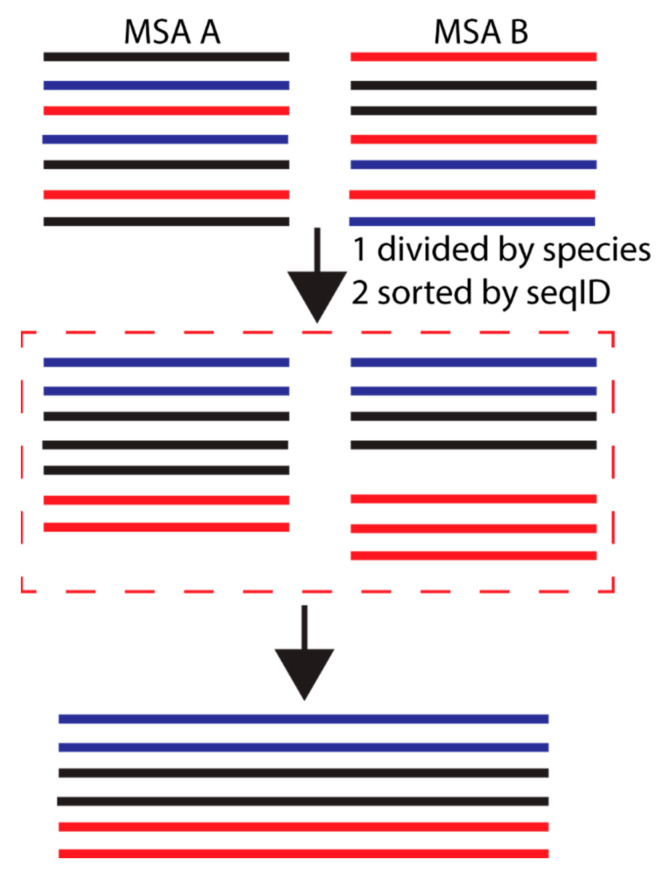
The flowchart of the phylogeny information-based method.

Stage 2. There are five steps in the cpxMSA construction based on the protein monomer sequence and species similarity search. First, download the taxonomy database from the National Center for Biotechnology Information (NCBI) public database. Secondly, compare the multiple sequence alignment information MSA_A and MSA_B of sequence A and sequence B in Stage 1 with the taxonomy database, respectively, to obtain the species information of the proteins in MSA_A_phy and MSA_B_phy, respectively. Third, rank the similarity of proteins and query sequences in each species in MSA_A_phy and MSA_B_phy from high to low. Fourth, let $P_{1},P_{2},\cdots ,P_{m}$ be the species-specific proteins in MSA_A_phy sorted by sequence similarity, and $Q_{1},Q_{2},\cdots ,Q_{n}\;$ be the species-specific proteins in MSA_B_phy ranked by sequence similarity. Then, connect $P_{i}$ with $Q_{i}$, where i≤min(m,n). Finally, according to the species comparison result, the two monomer multi-sequence comparisons are concatenated to obtain the species-based multi-sequence comparison result of the protein complex (see Figure 6).

Stage 3. The main points of the process (according to the protein interaction network to build cpxMSA) are as follows: (i) Download the protein interaction information (STRING linker) and protein interaction sequence information (STRING database) from the protein interaction network database (STRING version 10.5v: https://cn.string-db.org/) of the public database. (ii) Use the multiple sequence alignment HHblits program to search for the protein interaction sequence information (STRING) of sequence A and sequence B, respectively, and obtain the multiple sequence alignment information MSA_stringA and MSA_stringB, respectively. (iii) According to the protein interaction information (STRING linker), determine whether any two proteins, protein i and j in the MSA_stringA and MSA_stringB, have interactions. If there is an interaction, connect the two. In summary, according to steps (i)–(iii), construct an interaction-based multiple sequence alignment (MSA) of the protein complexes.

### 3.2. Selection of the Protein Complex Multiple Sequence Alignment Method

The number of effective sequences of the protein complex multiple sequences alignment (*Necs*):

(1) $$Necs=\frac{1}{\sqrt{L}}\overset{N}{\underset{i=1}{\sum }}\frac{1}{1+\sum_{j=1,i\neq j}^{N}\delta \left(S_{i,j}\geq 0.8\right)}$$

(2) $$S_{i,j}=\frac{2}{\frac{1}{S_{iA,jA}}+\frac{1}{S_{iB,jB}}}$$

(3) $$\delta \left(S_{i,j}\right)=\{\begin{array}{c} 1,\;if\;S_{i,j}\geq 0.8\\ 0,if\;S_{i,j}\leq 0.8 \end{array}$$

where *L* is the length of the query protein complex and *N* is the number of sequences in the protein complex multiple sequence alignment (MSA). $S_{iA,jA}$ is the sequence identity between chain *A* in sequence i and chain *A* in sequence j. $S_{iB,jB}$ is the sequence identity between chain *B* in sequence i and chain *B* in sequence j.

Selection of protein complex multiple sequence alignment method: First, calculate the number of effective sequences in the multiple sequence alignment of the protein complex based on genomic distance in stage 1. Secondly, if the number of sequences in the multiple sequence alignment in stage 1 meets the requirements, the sequence alignment in stage 1 is used as the input in the step of removing redundant sequences. Otherwise, combine the multiple sequence alignment in step 1 with the multiple sequence alignment based on the species category in stage 2, and calculate the number of effective sequences. Thirdly, if the number of valid sequences after the merging of stage 1 and stage 2 meets the condition, the merging result is used as the input of the redundant sequence step. Otherwise, combine the multiple sequence alignments based on the protein interaction network in stage 1, stage 2, and stage 3 as the input to the step of removing the redundant sequences.

## 4. Conclusions

We developed an open-source pipeline, cpxDeepMSA, to provide a cpxMSA algorithm that is high-quality, large-depth, and provides a wide range of sequence sources and strong generalization abilities. cpxDeepMSA was proposed to solve the shortcomings of low-quality cpxMSA results due to a single database and low search depth. The advantages of cpxMSA by cpxDeepMSA are as follows: (i) It increases the depth of MSA. The depth of MSA, not just using the single search algorithm or database to align, can also judge according to the number of valid sequences in the MSA results from the previous layer. (ii) The proposed method enhances the generalization ability by using different protein monomer databases and three different monomer MSA strategies (genomic distance, phylogeny information, and STRING interaction network) to join the monomer MSA results in protein complexes. The online server and the standalone program of cpxDeepMSA are freely available at https://zhanggroup.org/cpxDeepMSA/ (accessed on 1 July 2022).

## Figures and Tables

**Table 1:** Inter-protein contact prediction precision on the PDB100 database by trRosettaX. Bold font indicates the highest value in each category.

MSA	L/5	L/10	L/20	L/50	50	20	10	5
Genomic-based	0.273	0.325	0.372	0.414	0.302	0.365	0.397	0.424
Phylogeny-based	0.353	0.430	0.499	0.564	0.394	0.485	0.538	0.577
STRING-based	0.210	0.253	0.295	0.333	0.228	0.282	0.316	0.338
cpxDeepMSA	0.398	0.491	0.572	0.645	0.449	0.560	0.629	0.673

**Table 2:** Inter-protein contact prediction precision (%) on Baker’s data.

Predictor	MSA	L/5	L/10	L/20	L/50	50	20	10	5
CCMpred	RF MSA	0.012	0.007	0.006	0.009	0.010	0.006	0.004	0.007
	cpxDeepMSA	0.137	0.214	0.306	0.377	0.242	0.333	0.385	0.400
tRosettaX	RF MSA	0.340	0.416	0.462	0.535	0.406	0.461	0.519	0.556
	cpxDeepMSA	0.334	0.418	0.487	0.565	0.410	0.494	0.552	0.578

## Data Availability

Not applicable.

## Abbreviations

PPI	protein–protein interaction
MSA	multiple sequence alignment
cpxMSA	protein complex multiple sequence alignment
Necs	effective sequences of the protein complex multiple sequences alignment
